# Forest productivity mitigates human disturbance effects on late-seral prey exposed to apparent competitors and predators

**DOI:** 10.1038/s41598-017-06672-4

**Published:** 2017-07-25

**Authors:** Daniel Fortin, Florian Barnier, Pierre Drapeau, Thierry Duchesne, Claude Dussault, Sandra Heppell, Marie-Caroline Prima, Martin-Hugues St-Laurent, Guillaume Szor

**Affiliations:** 1 Chaire de Recherche Industrielle CRSNG-Université Laval en Sylviculture et Faune, Centre d’étude de la forêt, Département de Biologie, Université Laval, Québec, Québec, G1V 0A6 Canada; 2 Chaire de recherche industrielle CRSNG – Université du Québec en Abitibi-Témiscamingue et Université du Québec à Montréal en aménagement forestier durable, Centre d’étude de la forêt, Département des sciences biologiques, Université du Québec à Montréal, Montréal, Canada; 3 Département de mathématiques et de statistique, Université Laval, Québec, G1V 0A6 Canada; 4 Direction de la gestion de la faune du Saguenay-Lac-St-Jean, Ministère des Forêts, de la Faune et des Parcs (MFFP), Jonquière, Canada; 5 Direction de la gestion de la faune de la Côte-Nord, MFFP, Baie-Comeau, Canada; 6 Département de Biologie, Chimie et Géographie, Centre d’étude de la forêt, Centre d’Études Nordiques, Université du Québec à Rimouski, Rimouski, Canada; 7 Direction de la gestion de la faune du Nord-du-Québec, MFFP, Chibougamau, Canada

## Abstract

Primary production can determine the outcome of management actions on ecosystem properties, thereby defining sustainable management. Yet human agencies commonly overlook spatio-temporal variations in productivity by recommending fixed resource extraction thresholds. We studied the influence of forest productivity on habitat disturbance levels that boreal caribou – a threatened, late-seral ungulate under top-down control – should be able to withstand. Based on 10 years of boreal caribou monitoring, we found that adult survival and recruitment to populations decreased with landscape disturbance, but increased with forest productivity. This benefit of productivity reflected the net outcome of an increase in resources for apparent competitors and predators of caribou, and a more rapid return to the safety of mature conifer forests. We estimated 3-fold differences in forest harvesting levels that caribou populations could withstand due to variations in forest productivity. The adjustment of ecosystem provisioning services to local forest productivity should provide strong conservation and socio-economic advantages.

## Introduction

Primary production determines multiple aspects of ecosystems, including the services that they provide, their resilience, and the strength of food-web interactions^1, 2^. For instance, predation tends to be a more dominant driving force in high- rather than low-productivity systems^1^. Food-web complexity, however, can modify the effect of primary production on top consumers^3, 4^. A rise in productivity can increase the competition between species in the top trophic level, leading to a net decrease in their overall abundance and their influence on food-web functioning^3^. Alternatively, a rise in productivity can increase the abundance of a particular prey species, leading to a numerical response by its predator with negative effects on other sympatric prey^3, 5^. Such an effect that prey species can exert on one another through a shared enemy – i.e., their “apparent” competition (sensu^4^) – can even result in population extirpation^6^. Whilst most of these theoretical predictions of trophic interactions reflect equilibrium solutions, natural systems often exhibit complex transient dynamics that can last for decades^7^. Few studies have examined how productivity alters the strength of top-down and bottom-up effects over time in dynamic systems^8^, such as those driven by habitat disturbances.

Species tend to experience the same disturbances differently, due to interactions between adaptive traits, landscape features, and the spatio-temporal scales at which they react to environmental drivers^9^. The current state and the path of ecological succession can thus favour some species over others^10^. An increase in primary production could amplify this asymmetry, given that productivity can alter the trajectory of plant succession and, subsequently, the relative intensity of top-down and bottom-up forces^3^. In addition to the trajectory, productivity can alter the resilience of ecosystems^7, 11^, by either increasing or decreasing the speed of recovery^12–15^. The net outcome on food web dynamics remains poorly documented despite previous work showing that primary productivity can influence both the asymmetry of the effect that a disturbance can impose on individual species (e.g., the higher productivity in southern than in northern boreal forests has a stronger positive impact on food available to moose, *Alces americanus*, than to boreal caribou, *Rangifer tarandus caribou*, during early stages of ecological succession^16^) and the duration of this effect by influencing the speed of ecological succession (e.g., the post-fire development of a dense forest canopy occurs more rapidly in more productive environments, which, for example, can induce a faster decline in species diversity^17^). Yet this information is necessary to preserve and manage biological systems, and to anticipate the availability of ecosystem provisioning services.

Our study focuses on a boreal ecosystem that is occupied by boreal caribou, moose, grey wolf (*Canis lupus*), and black bear (*Ursus americanus*), and where logging is the main industrial activity. The boreal forest is one of the dominant biomes on Earth, and two-thirds of its area is currently managed^18^. Boreal caribou populations are threatened with extinction over most of the biome^19^, a situation that logging activities have tended to exacerbate by impacting food-web interactions^20^. Boreal caribou populations are under top-down control^21–23^, and black bears and wolves are the two predators involved in eastern Canada^24, 25^. Black bears can be the dominant predator of caribou calves^24, 26^, their density is highest in early stages of forest succession^27^, and they are particularly attracted to areas of high primary production^28, 29^. Wolves consume both caribou calves and adults^24, 30^. Wolf density covaries with moose density^31^, making moose a strong apparent competitor of caribou^32, 33^. The carrying capacity and size of moose populations increase with deciduous vegetation availability^34, 35^, and as a result, moose (and thus wolves^31^) are most abundant in landscapes largely comprised early-seral forests^27, 36^, such as those impacted by logging activities. Forest productivity further increases carrying capacity of moose populations^35^, and moose-wolf interactions are even more closely associated with cut-blocks where primary productivity is high^16^. The increased local moose population could trigger a numerical response by the wolf population, with negative consequences for the caribou^5, 37^, as as long as their preference for alternative prey species do not draw wolves away from prime caribou habitat^38^. Given the impact of logging on boreal caribou populations, current recommendations that serve to aid this ungulate’s recovery include the restriction of total disturbance (i.e., fire- and human-induced) to 35% of the landscape, because this threshold yields a 60% probability for a local caribou population to be self-sustaining^22^. While the threshold is recommended for the entire Canadian boreal forest, its effect on caribou populations may translate into geographic differences of population parameters^39^. For example, boreal caribou populations are established in portions of the Canadian boreal forest where extensive and recurrent fires can keep disturbance at levels exceeding 35%^22, 40^. Some populations thus appear able to withstand such relatively high disturbance levels. Population differences in the vital rates of caribou could in part be driven by spatial variations in primary productivity^16^. On one hand, the growth of deciduous vegetation that is consumed by moose should achieve greater biomass in more productive forests^16, 35^, which should translate into higher wolf densities^31^. On the other hand, secondary succession in a more productive environment should develop more rapidly towards closed-canopy conifer forests^7, 18^, a stand type providing greater benefit to caribou than to other members of the food web. The net outcome of these two conflicting trends on caribou populations remains unclear. This information has both conservation and socio-economic relevance: the use of a fixed threshold could be too permissive in some areas to protect local caribou populations, or too restrictive in other areas, given timber harvesting objectives that optimise wood harvest while maintaining an “acceptable” risk of decline in the local caribou population.

This research assesses the interplay between boreal forest productivity, ecosystem resilience, and the persistence of caribou populations. We then use this information to infer the opportunity for provisioning services (wood extraction) in boreal forests. First, we established the link between forest productivity and secondary succession. Second, we determined the effects of forest productivity on the potential resilience of caribou populations to habitat disturbances. On this basis, we estimated the level of habitat disturbance that caribou populations could withstand, given the local forest productivity experienced by our six study populations. The level of disturbance then informs on spatial variations in the opportunity for provisioning services (wood extraction) in forested landscapes that are inhabited by the threatened boreal caribou.

## Results

**Figure 1:**
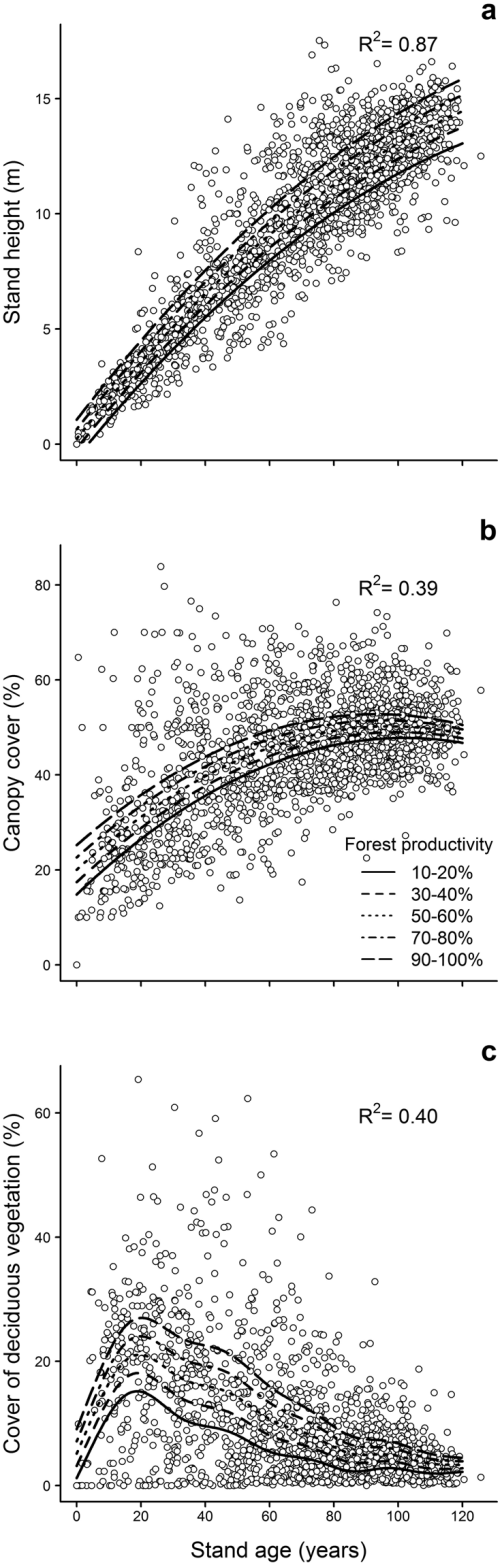
(**a**) Stand height, (**b**) total canopy cover, and (**c**) canopy cover comprised of deciduous vegetation, as a function of their age for five categories of proportions of potentially productive stands (forest productivity) in eastern Canadian boreal forest. The lines are estimated from the models that are presented in Supplementary Tables S1 and S2, and, accordingly, the influence of productivity on all three stand attributes varies significantly with age.

Our analysis of vegetation changes during secondary succession showed that the proportion of potentially productive forest stands (PP_productive_forest_stands_) was positively linked to the resilience (i.e., ability to recover from a perturbation^14^) of boreal forest ecosystems (Supplementary Tables S1 and S2). After controlling for time-since-disturbance, we found that stands grew faster and reached greater heights in areas with higher PP_productive_forest_stands_, while also reaching a closed canopy condition more rapidly (Fig. 1). For example, a forest with 10-20% potentially productive stands would reach its maximum canopy cover >20 years later than a forest with 70–80% of those stands (Fig. 1b). While the maximum cover of deciduous trees occurred ~20 years after a landscape had been disturbed, regardless of forest productivity, areas with higher PP_productive_forest_stands_ become richer in deciduous vegetation. A forest that was characterised by 10–20% potentially productive stands was typically occupied by ~15% fewer deciduous trees than a forest with 70–80% potentially productive stands (Fig. 1c).

**Figure 2:**
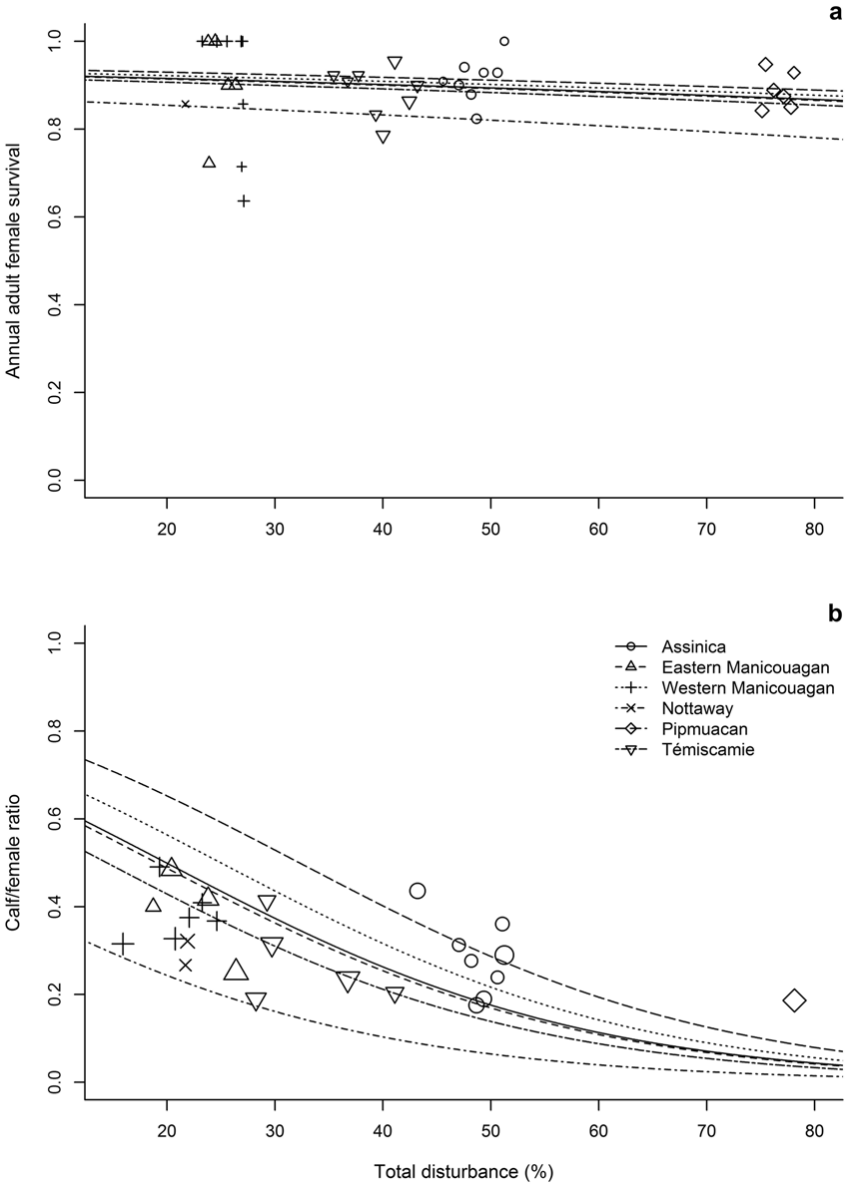
Variation in weighted estimates of (**a**) female survival and (**b**) calf/female ratio (the recruitment index) as a function of total disturbance within six ranges of boreal caribou in eastern Canadian boreal forest. Lines are predicted trends and symbols are the actual values, with the size reflecting the number of observed females during each year of survey (range: 6–22 females for survival; range: 50–252 females for recruitment index).

Both adult female survival and recruitment (ratio calf/female) to caribou populations decreased with the level of total landscape disturbance but increased with PP_productive_forest_stands_ (Table 1). In a landscape that has been disturbed by 35%, for example, the statistical model predicts annual survival of 0.81 and 0.91 for adult females of populations that were located respectively in the least productive (Nottaway: 21% potentially productive stands) and the most productive (Pipmuacan: 85% potentially productive stands) forests (Fig. 2a). In the same two landscapes, the calf/female ratio would be 0.14 for the population that was located in the range with the lowest PP_productive_forest_stands_, and 0.45 in the range where PP_productive_forest_stands_ was highest (Fig. 2b).

**Table 1:** Parameter estimates (coefficients, standard errors, and 95% confidence intervals) of mixed-effects models describing the effects of total proportion of disturbances and proportion of potentially productive stands (squared, in the case of recruitment) on the annual survival of adult female caribou, and on recruitment (i.e., calf/female ratio observed during aerial surveys) to six boreal caribou populations distributed over 380,000 km^2^ of eastern Canadian boreal forest (*n* = 24 surveys among the six populations). Overall, observed values ranged between 0.22 and 0.78 for total proportion of disturbances and between 0.04 and 0.85 for proportion of potentially productive stands.

Covariate	Adult survival		Recruitment	
	β ± SE	95% CI [lower: upper]	β ± SE	95% CI [lower: upper]
Intercept	1.67 ± 0.29	[1.10: 2.27]	−0.15 ± 0.59	[−1.30: 1.00]
Total disturbances	−0.77 ± 0.29	[−1.35: −0.20]	−5.05 ± 0.14	[−5.35: −4.76]
(Potentially productive stands)²	—	—	2.41 ± 1.22	[0.04: 4.80]
Potentially productive stands	1.22 ± 0.54	[0.13: 2.26]	—	—

**Figure 3:**
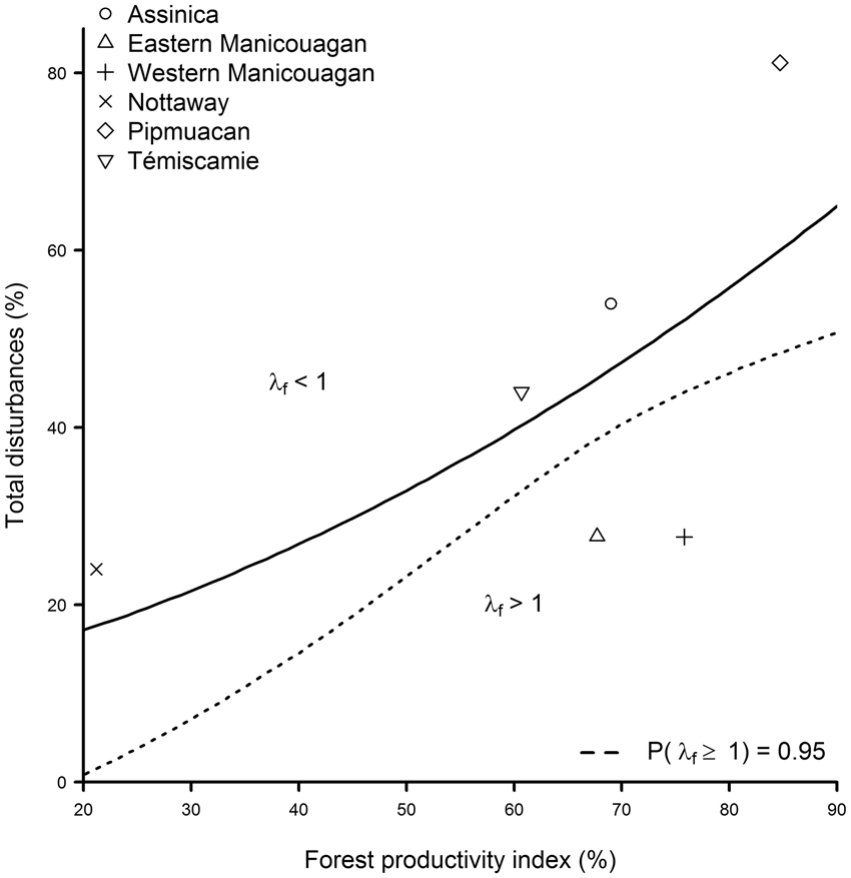
Combination of total disturbance (fire and logging) and the proportion of potentially productive stands (the forest productivity index) for which the annual rate of change in the female population (*λ*_*f*_) is equal to 1 (solid line), with the lower limit yielding *λ*_*f*_ ≥ 1 at least 95% of the time. The population tends to increase (*λ*_*f*_ > 1) below the line and decline (*λ*_*f*_ < 1) above it. Symbols represent the combination for individual populations, with respect to the observed combination of disturbance during their last year of monitoring.

Based on the relationships between vital rates, total forest disturbance and PP_productive_forest_stands_, we estimated the level of disturbance that the different caribou populations should be able to withstand. On average for our six populations, 44 ± 14% (mean ± SD) of disturbances in the landscape should yield a stable population (*λ*_*f*_ = 1; Supplementary Table S3), with predicted levels differing amongst populations from 18–60%, due to spatial variation in PP_productive_forest_stands_. These average levels, however, are associated with statistical uncertainty such that to be confident that, 95% of the time, the local population would be at least stable over the short-term (*λ*_*f*_ ≥ 1), the disturbance level should average 34 ± 14%, ranging amongst populations from 2 to 49%. Of the six populations that were studied, four were established in a range where the level of disturbance for population viability was already exceeded (Fig. 3).

## Discussion

Based on 10 years of intensive monitoring of six boreal caribou populations ranging over 380,000 km^2^ of boreal forest, we provide empirical evidence that increased resilience of boreal ecosystems associated with high forest productivity can yield net benefits to a late-seral, top-down driven ungulate despite an increase in food resources favouring its apparent competitor and predators. Our study brings into play two opposing ecological forces that are acting on caribou populations: (1) productivity increases food for apparent competitors and predators in early-stages of forest succession, and (2) productivity increases the resilience of boreal forest ecosystems, such that conifer stands providing protection to caribou come back faster in more productive forests.

**Figure 4:**
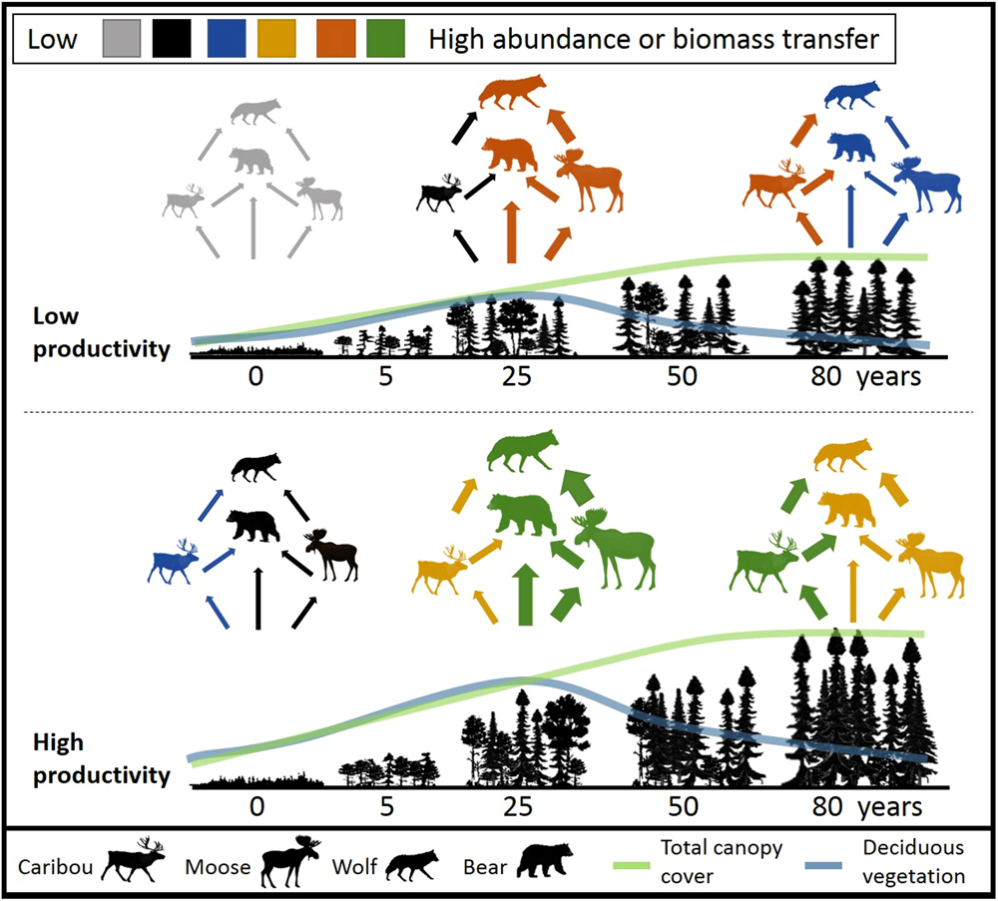
Changes in forest stand characteristics during the first 80 years of secondary succession in eastern Canadian boreal forest, together with a potential scenario of changes in trophic interactions that is consistent with the higher growth of caribou populations observed in more productive boreal forests. Change in animal size reflects how the abundance of a given species varies over time, whereas arrow size is proportional to trophic interaction strength. The illustration indicates that in 25-year-old stands, moose have access to more deciduous vegetation in high than low productivity forests, which results in higher moose density in landscape largely comprised of these stands, and subsequently in higher grey wolf density. Likewise, black bears have access to more grasses, forbs and berries in 25-year-old stands found in more productive landscapes, thereby leading to high predation rates on calves and, therefore, to relatively low recruitment in productive forests that are largely comprised of these early-seral stands. Ultimately, the faster succession towards relatively safe forests that are largely comprised closed-canopy conifer stands would be largely responsible for the relatively high growth of boreal caribou population in high- than low-productive forests.

Our analysis confirms that the key relationships expected between time-since-disturbance, forest productivity (PP_productive_forest_stands_) and deciduous vegetation availability are indeed present in the study area (Fig. 4). Predators and apparent competitors should therefore be most abundant in broadly disturbed landscapes (i.e., high proportion of <40- to 50-year-old stands given our disturbance criteria), with negative consequences expected on caribou populations^22, 41^. Accordingly, we found that adult survival and recruitment to caribou populations decreased with the level of landscape disturbance. However, the impact of productivity on caribou demography is not as easy to anticipate in such complex, transient systems.

Forest productivity should have an asymmetrical impact on the two apparent competitors of this food web. Boreal caribou feed largely on shade-intolerant, slow-growing terrestrial lichens, especially during winter^42^. By contrast, moose favour deciduous vegetation that becomes most abundant in early stages of boreal forest succession^34^. The positive link between forest productivity and deciduous vegetation during early stages of forest succession (Figs 1 and 4) should therefore have a larger impact on food availability for moose than for caribou. Also, primary productivity influences interaction strengths in plant-prey-predator systems following a combination of non-linear inter-species relationships e.g., numerical and functional responses, and frequency-dependent prey selection by the predator^4, 5, 43^, and habitat-animal spatial relationships^16, 44, 45^. In this context, we can consider the typical situation where an increase in deciduous vegetation is followed by an increase in moose abundance that triggers a numerical response by the wolf population. Wolves should then increase their focus on moose as they should be more abundant than caribou^44^, especially in a highly productive forest (Fig. 4). The consequence should be a stronger increase in predation rate for moose than for caribou, as can be estimated from the model provided in^44^, with species-specific habitat selection provided in^16^. Moreover, the impact of forest productivity on the demography of boreal caribou depends upon the relationship between forest resilience and productivity. This is because stands of the same age have different characteristics if they are in landscapes with a high rather than a low forest productivity index (Fig. 1).

We showed that an increase in our forest productivity index is linked to a faster recovery of mature boreal forests, as expected from the resilience-productivity hypothesis^13^. As previous reports have shown^46^, we found that forest stands grew taller and reached the closed-canopy cover stage more rapidly following disturbance when they occupy an environment with a high productivity index. By accelerating the return of landscape features that are safe for boreal caribou i.e., closed-canopy conifer forests^47, 48^ by ca. 20 years, and by more rapidly decreasing the availability of vegetation that is consumed by black bears (Fig. 4), areas with a high productivity should decrease the window of high predation risk for caribou. Consistently, our analysis of vital rates of caribou populations revealed that the positive impact of forest productivity on the resilience of forest ecosystems provides net benefits to caribou populations. Although our explanation for this result is based upon mechanisms derived from decades of research on the caribou-moose-bear-wolf system across North American boreal forests, including the study area, it remains based on inductive reasoning. What is clear and non-speculative, though, is our demonstration that late-seral prey can achieve higher survival and recruitment rates in landscapes that are characterised by higher forest productivity, even though these landscapes provide more food to their apparent competitors, at least at some point during ecological succession. This result is significant because our understanding of food-web dynamics following environmental changes is largely based on equilibrium responses of food webs determined from theoretical models^3, 49^; our empirical assessment thus is one of the few that reveals how primary productivity affects animal populations in systems under complex transient dynamics through an influence on trophic-interaction strengths during ecological succession.

Because caribou and moose have largely different diets, their food web can be represented by parallel energy pathways (sensu^49^) connected at a higher level by their shared predators. Under these conditions, factors (e.g., energy availability) that increase the density of an apparent competitor can result in the decrease – even the extirpation – of the other prey^50^. Indeed, the typical expectation for caribou-moose-wolf systems is that a change in landscape conditions favouring moose will negatively affect the local caribou population^33^. Our findings are at odds with this prediction, which suggests that the impact of forest productivity on the resilience of boreal forest largely drives the overall consequences of forest harvesting on caribou populations. Furthermore, our assessment of the interplay between forest productivity and caribou demography provides key information for animal conservation in landscapes that are also expected to provide ecosystem-provisioning services. Indeed, we found that the threatened boreal caribou should be affected by lower levels of disturbance when occupying less productive forests. Commercial timber harvesting should be adjusted to local primary production, given our finding that cut-blocks need to occupy a smaller portion of the landscape in areas with a lower productivity index to maintain the viability of caribou populations. This finding implies that the fixed disturbance threshold currently recommended for the management of caribou habitat^22^ could have both conservation and socio-economic drawbacks. The population located in the least productive forest (Nottaway) should face a high risk of declining (or being a population sink), even when the percent of total disturbance is nearly half the 35% threshold that is currently advocated by the Canadian government^22^. Conversely, populations that are located in the most productive forests of the study area should be able to withstand greater disturbance than the 35% threshold. In our case, however, total disturbance already exceeded the level that four of the six study populations should be able to withstand (Supplementary Table S3), even when considering that some are established in forests with high productivity indices. Our findings with regards to the forest productivity-disturbance-caribou population persistence relationship thus corroborate the importance of adopting range-specific boreal caribou conservation planning guidelines in managed forest landscapes^39^. Hence, this relationship provides an element that could be used to refine Environment Canada’s recovery plan^22^.

This research demonstrates that late-seral species, such as boreal caribou, could benefit from the increase in primary productivity that is expected in many areas of the boreal forest^51^, including in eastern Canada^52^. Our predictions on demographic responses of the boreal caribou to disturbance levels are made within the context of conventional even-aged management with short-rotation using clear-cutting as the main timber harvesting practice. In many parts of boreal caribou’s range, however, the rotation period between successive forest harvest events is shorter than what is observed under natural disturbance regimes^53^. For example, the mean fire return interval exceeds 250 years in the range of Manicouagan caribou populations^54^, which drastically exceeds potential harvest rotation cycles of 50–60 years^55^. Ecologists have proposed alternatives (e.g., ecosystem-based management) to short-rotation clear-cutting that consider the variability of natural disturbance regimes through extensive use of longer rotations, partial cutting, and variable retention^56^. Whereas such approaches are more likely to maintain biodiversity^57, 58^, they have yet to be implemented extensively and the socio-economic pressure still promotes clear-cutting as the main harvesting practice in the boreal forest. It was thus important to forecast our results within the conventional even-aged short-rotation management framework. Climate change in the Canadian boreal forest may, however, accelerate the implementation of ecosystem-based forestry practices to ensure forest resilience in the future^18^.

Current guidelines define a disturbed area as one that has been burned or logged in the past 40–50 years^22, 59^, which implies that the 65% of undisturbed forest within a caribou range, at least in principle, could be comprised of 51-year-old stands. Such a concentration of rather young stands would create forest conditions approaching those where moose, wolves and black bears are generally most abundant^60^. Predation rates on boreal caribou could become correspondingly higher than those that are currently observed because lag effects in population dynamics are such that predators can remain high a few years after the decline of their main prey^61^. Fast rotations between successive harvests are likely to affect caribou populations negatively, and to decrease the level of total disturbance that populations can withstand. Likewise, our model’s predictions should hold as long as the logging schedule results in an age structure of harvested stands similar to the structure used in our analysis, which implies, for example, that harvested stands should not all belong to the 0–5 age class (NB, multiple years are needed to harvest the area occupied by a caribou population, see refs 62, 63). Given such uncertainties, we caution that the thresholds emerging from our study should not be seen as targets for the forest industry; rather, they should be viewed as disturbance levels where the situation for threatened boreal caribou could rapidly become critical^37^.

With the growth of human populations, there is increasing need for both extraction of natural resources and the protection of species that are faced with extinction. These two goals often conflict with one another. We have shown that forest primary production can influence trophic interactions during secondary forest succession in a way that mitigates the negative effects of cumulative disturbances on the viability of a late-seral ungulate species. This possibility reflects the positive outcome resulting from the contrasting forces of the increase in food resources for apparent competitors and predators, and the faster recovery towards the safety of closed-canopy mature forests. Still, there is always the risk that caribou populations may not be able to take advantage of the positive outcomes of forest productivity, given their continuing retreat towards northern, less productive forests. Also, boreal caribou appear to be more closely associated with muskegs^64^ in western Canada than in our study area^65^, a difference that could indicate that factors other than forest productivity may further alter how logging levels influence the vital rates of caribou populations across the boreal forest. For now, our study demonstrates that conservation biologists, forest managers, and industrial stakeholders can collectively benefit from a better understanding of local and regional drivers of threatened animal populations. In a world being altered at an increasing pace due to climate change and human activities, there are strong ecological, societal and economic advantages of forecasting change in biodiversity and natural resource availability accurately^66, 67^, and our study highlights the importance of considering how primary productivity impacts food-web interactions throughout the sequences of transient phases and associated lags in population responses that should characterise future ecosystem dynamics.

## Methods

**Figure 5:**
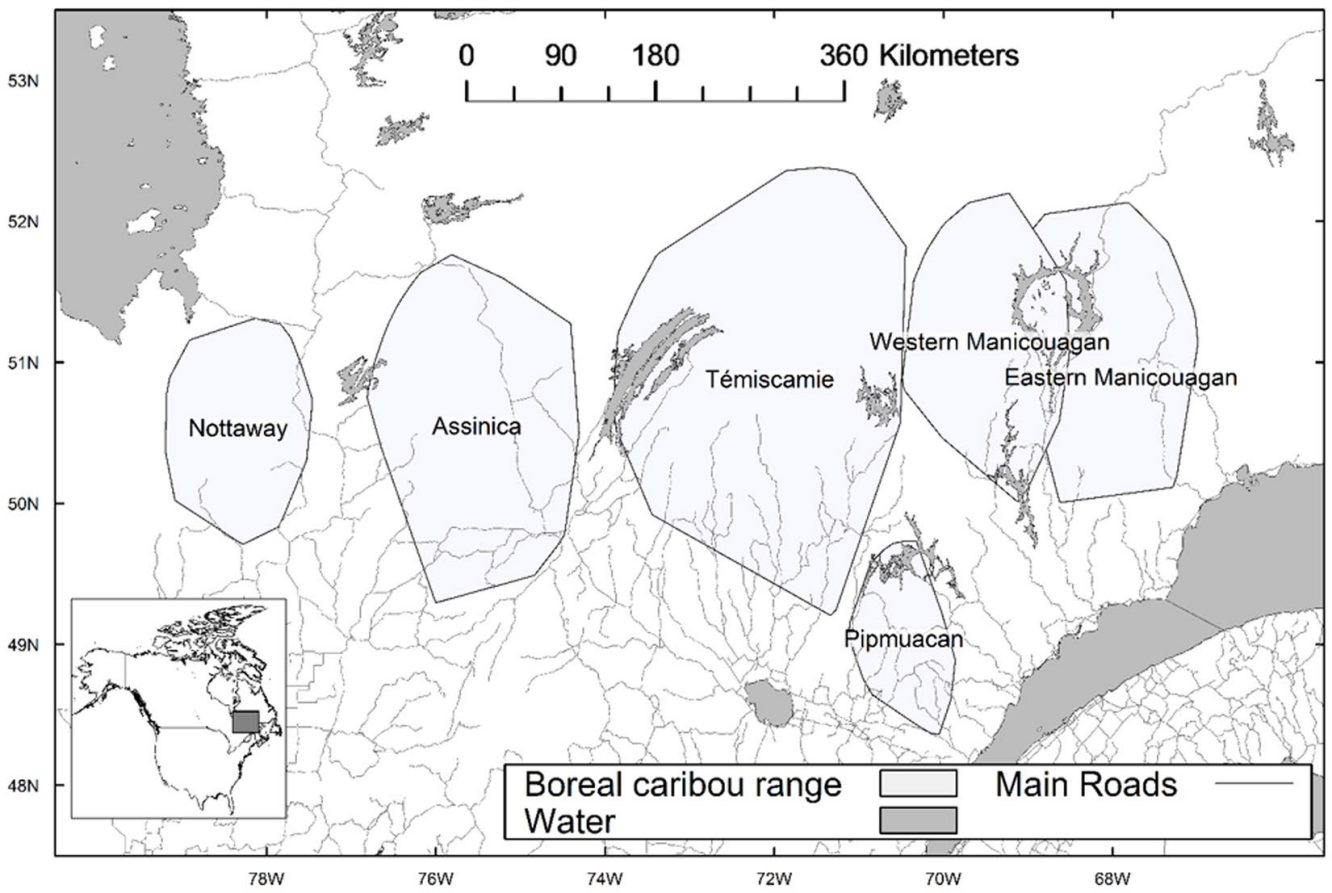
Location of six populations of boreal caribou that were monitored in eastern Canadian boreal forest. The figure was created in the R statistical environment^73^.

Our study area encompasses ~380,000 km^2^ of boreal forest in northeastern Canada. This area is dominated by stands of black spruce (*Picea mariana* [Mill.] BSP); balsam fir (*Abies balsamea* [L.] Mill.) and jack pine (*Pinus banksiana* Lamb.) are also common. Mean annual temperature varies between 2.1 and 5.5 °C, mean annual precipitation varies between 900 and 1100 mm, and elevation reaches 1150 m. Six populations of boreal caribou were present in our study area: Nottaway, Assinica, Témiscamie, Pipmuacan, and western and eastern Manicouagan (Fig. 5). A total of 186 female caribou were monitored with GPS collars between 2004 and 2014 in these populations.

Both captures and manipulations of study animals were approved by the Animal Welfare Committee (according to the guidelines of the Canadian Council on Animal Care) of the Université du Québec à Rimouski (certificates #36-0867 and #27-07-53), Université Laval (certificate #2008026-3) and of the Ministère des Forêts, de la Faune et des Parcs du Québec (hereafter referred to as MFFP; certificates #07-00-02, #04-005, #06-00-27, #07-00-04, #11-03, #12-03, #12-07, #13-09 and #14-05).

### Forest stand characteristics

We evaluated forest productivity in caribou population ranges using a proxy based on the proportion of potentially productive stands (PP_productive_forest_stands_) in ecological districts, as estimated by^68^. In their analysis, stands were considered as productive based on two thresholds of minimum harvesting volume: a merchantable volume greater than 50 m^3^/ha (stand level), and a harvested stem volume greater than 70 dm^3^ (stem level). PP_productive_forest_stands_ reflects the potential for stand regeneration, following natural or anthropogenic disturbance, and it is calculated from biophysical attributes of forest stands^68^ for more information. We estimated the relative index of forest productivity by geo-referencing Fig. 4B of ref 68, and then calculated, for each caribou population, the average PP_productive_forest_stands_ amongst the districts (see ref. 68) that were included in the population’s range, weighted by their relative area. This range corresponded to the 99% minimum convex polygon that was estimated from all radio-collared individuals of a given population.

Mean stand age, mean stand height, proportion of canopy cover, and proportion of deciduous trees were retrieved from the National Forest Inventory of Canada^69^, a Geographic Information System of 250-metre resolution. Natural and anthropogenic disturbances were extracted from digitised ecoforest maps from the 4^th^ inventory that was produced by the Ministère des Forêts, de la Faune et des Parcs du Québec (minimum mapping unit size of 4 ha for forested polygons and 2 ha for non-forested areas). For areas that were not covered by the ecoforest maps, we used the Canadian National Fire Database (CNFD), which was provided by the Canadian Forest Service to map burned areas until 2014. The proportion of total disturbances was calculated by incorporating roads, cuts < 50-years-old^59^, and natural disturbances < 40-years-old^70^. The proportion of total disturbance was updated every year to account for ongoing industrial activities. Following^22^, we also applied a 500-metre buffer (i.e., zone of influence) around anthropogenic disturbances. We estimated the proportion of total disturbance by considering overlapping areas only once (e.g., overlap between the 500-metre buffers of a road and a cut). The proportions of potentially productive stands and total disturbance were estimated for each population’s range.

### Aerial survey and sex ratio assessment

Aerial surveys were conducted periodically for each herd from late February to late March, between 1999 and 2014. The survey technique should yield an 85% probability of detection^71^. First, areas were systematically surveyed by aircraft to locate caribou track networks. The following day, a helicopter flew the networks at a lower altitude to count and classify caribou as calves, adult males and adult females, according to body size, antler morphology, and presence of a vulvar patch. When the sex could not be determined, caribou were classified as either adults or calves. Misclassification of calves is unlikely, as they are morphologically different from adults, and they tend to stay next to their mothers. In addition to aerial surveys, between 6 and 22 caribou/year were tracked with GPS-collars for each population, and the composition of their group was determined each time they were monitored. When it was impossible to determine the sex of adults, we used the female-to-male sex ratio to classify unknown adults. When fewer than 10% of adults were unclassified, we used observed numbers of females and males to calculate sex ratios; otherwise, we followed^72^ by assuming 65% were females and 35% were males (i.e., 0.54 sex ratio). This assumption is consistent with the average sex ratio of individuals for which the sex was successfully determined during aerial surveys (699 caribou: 246 males and 453 females). Calf/female ratios were estimated for each year, based on available surveys or monitoring information.

### Statistical analysis

#### Productivity

We determined how the structural development of forest stands varied with our index of forest productivity (PP_productive_forest_stands_). We randomly selected 2000 points within the study area. For each point, we extracted four stand characteristics from the National Forest Inventory of Canada maps^69^, which corresponded to mean characteristics of forest stands within a pixel size of 250 × 250 m, i.e., mean stand age, mean stand height, proportion of canopy cover, and proportion of deciduous vegetation. Because canopy cover was calculated based on total pixel area, including treeless areas, we corrected it by dividing the given canopy cover with the proportion of the area that was covered by trees. This allowed us to obtain a value corresponding to the mean proportion of ground covered by tree canopy in forest stands within this 250 × 250 m area. The proportion of deciduous vegetation was also corrected to take into account the non-vegetated area of the pixel. Pixels without vegetation or without trees were removed from all analyses. We used linear models to determine how stand height and canopy cover varied with PP_productive_forest_stands_ and stand age. We added a quadratic term to account for possible non-linear effects. We then used a generalised additive model to identify the functional relationship between the proportion of deciduous trees and forest productivity and stand age. Statistical models further included an interaction term between stand productivity and age. Statistical analyses were performed using *lm* (linear models) and *gam* (generalised additive model) functions in the R statistical environment^73^.

#### Survival rate

Yearly survival rates were estimated for radio-collared adult females, using the staggered-entry Kaplan–Meier nonparametric estimator^74^. We modelled annual survival rates between 1 April and 31 March, to correct for the fact that individuals were not all captured on the same day. The Kaplan-Meier nonparametric estimator requires tracking individual status over time; hence, the status of individuals – dead or alive – was determined for each telemetry location. For every year, we estimated survival rate based on caribou belonging to a population for which at least six individuals were followed that year (Assinica: 15.1 ± 2.5 ind/year [N = 8 years]; Manicouagan East: 17.6 ± 2.5 [N = 5]; Manicouagan West: 9.1 ± 1.6 [N = 8]; Nottaway: 7 [N = 1]; Pipmuacan: 18.3 ± 2.3 [N = 6]; Temiscamie: 17.1 ± 5 [N = 8]). When an animal stopped being monitored for reasons other than predation-related mortalities (e.g., collar removal, mortality from hunting or accident), it was not considered as dead but simply right-censored, and it was therefore removed from the at-risk pool after its last “alive” location^74^.

The relationship that the proportion of total disturbance and forest productivity has with adult female survival was estimated using a generalised linear mixed model. We accounted for the possible non-independence between survival values for animals within the same population by using a random intercept for individual populations. As the total number of monitored females varies from year to year and influences the precision of the yearly survival rate estimates, we weighted our observations with this number for each survival value. Statistical analyses were performed using the *glmer* function in R, with 95% confidence intervals of the model coefficients being determined from a parametric bootstrap (*n* = 10,000 iterations) that was estimated with the *confint.Mermod* function^75^.

#### Recruitment

Recruitment to a caribou population was estimated from calf/female ratios that were determined during aerial surveys where at least 50 individuals were observed. Using a generalised linear mixed model with a binomial distribution, we estimated the link that the proportion of total disturbance and forest productivity shared with calf-to-adult female ratios. Forest productivity was transformed ([PP_productive_forest_stands_]^2^) to linearize the relationship. Non-independence between recruitment values for a given population was accounted for by using a random intercept for individual populations. Because the total number of observed females could influence the precision of recruitment estimates, we weighted our observations with the corrected number of females for each recruitment estimate.

Because surveys took place from February to March, i.e., relatively close to the next May-June calving season^24^, the number of observed calves reflected the survival and reproduction of adult caribou females during the previous year, and the survival of their calves over ~9 months. The disturbance level that was associated with a given survey was therefore the proportion of total disturbance observed the year preceding that survey. If we were to consider the total disturbance for the ongoing year instead, we would overestimate the disturbance level that the caribou experience, because some areas were yet to be disturbed. Statistical analysis of caribou recruitment was conducted with the *glmer* function, and the 95% confidence intervals of model coefficients were estimated with a parametric bootstrap (*n* = 10,000 iterations) that were conducted with the *confint.Mermod* function.

#### Finite annual rate of population change

Based on statistical models that had been previously developed (Table 1), we estimated *S*_*f*_ and *R* for a broad range of combinations between total disturbance (*D*) and forest productivity (*P*). We then identified the combinations yielding a finite annual rate of population change (*λ*) equal to unity, specifically for the female segment of the population (*λ*_*f*_). We focused upon females because they largely determine the dynamics of ungulate populations^76^. Following^77^, we estimated *λ*_*f*_ using: *λ*_*f*_ (*D*,*P*) = *S*_*f*_(*D*,*P*)/[1 – *R*_*f*_(*D*,*P*)], where *S*_*f*_(*D*,*P*) is the estimate of annual adult female survival for a combination of *D* and *P*, and *R*_*f*_(*D*,*P*) is the recruitment rate of females to the caribou population for the same combination. Assuming a sex ratio of 0.5 at birth, *R*_*f*_ was estimated from: *R*_*f*_ = (*R*/2)/[1 + *R*/2], where *R* is the calf/adult female ratio that had been determined from field surveys. We then simulated 10 000 values of *S*_*f*_(*D*,*P*) and *R*_*f*_(*D*,*P*) from their respective models for pairs of (*D*,*P*) values, and computed the corresponding *λ*_*f*_ (*D*,*P*). For each *P*, we then found the 95^th^ percentiles of all simulations of the effect of the *D* that yielded *λ*_*f*_ ≥ 1; this limit indicates that for a given forest productivity and given the uncertainty associated with the statistical model, we are 95% confident that the disturbance levels below this limit will yield *λ*_*f*_ ≥ 1.

### Data Availability

The datasets analysed during the current study are available in the Dryad repository, doi:10.5061/dryad.5jf43.

## Electronic supplementary material

Supplementary information
