# Supplementary material for: Forest productivity mitigates human disturbance effects on late-seral prey exposed to apparent competitors and predators
Source: Sci Rep. 2017 Jul 25;7:6370. doi: 10.1038/s41598-017-06672-4 (PMC5526934; doi:10.1038/s41598-017-06672-4)
Supplement: Supplementary file 1 — Supplementary information [file 41598_2017_6672_MOESM1_ESM.pdf]

Forest productivity mitigates human disturbance effects on late-seral prey exposed to apparent competitors and predators

By Daniel Fortin, Florian Barnier, Pierre Drapeau, Thierry Duchesne, Claude Dussault, Sandra Heppell, Marie-Caroline Prima, Martin-Hugues St-Laurent, Guillaume Szor

SUPPORTING INFORMATION

Supplementary tables

Table S1. Coefficients and their standard errors (SE), and associated *t*- and *P*-values for the linear models assessing the relationship of the proportion of canopy cover and stand height share as a function of stand age and proportion of potentially productive stands ($PP_{productive\_forest\_stands}$) in eastern Canadian boreal forests. Canopy cover: adjusted *R*-square: 0.394. Stand height: adjusted *R*-square: 0.871.

	Canopy cover				Stand height			
	Coefficient	SE	*t*	*P*	Coefficient	SE	*t*	*P*
Intercept	12.863	1.339	9.608	<0.001	-0.901	0.192	-4.744	<0.001
Age	0.657	0.036	18.508	<0.001	0.170	0.005	33.477	<0.001
Age^2	-0.0032	0.0002	-12.242	<0.001	-0.0005	0.00004	-13.056	<0.001
$PP_{productive\_forest\_stands}$	12.991	1.962	6.625	<0.001	2.074	0.281	7.390	<0.001
$PP_{productive\_forest\_stands}$ × Age	-0.069	0.027	-2.585	0.01	0.012	0.004	3.112	0.002

Table S2. Coefficients and their standard errors (SE), and associated *t*- and *P*-values of parametric coefficients, and estimated degrees-of-freedom (*edf)* and *F*- and *P*-value of smooth terms, for the generalised additive model (GAM) testing the effects of the total proportion of potentially productive forest stands ($PP_{productive\_forest\_stands}$) and stand age on the percentage of stands comprised of deciduous vegetation. Adjusted *R*-square: 0.395.

	% cover of deciduous trees					
	Coefficient	SE	*t*	*Edf*	*F*	*P*
Intercept	3.999	0.376	10.631	-	-	< 0.001
spline(Age)	-	-	-	8.329	14.75	< 0.001
$PP_{productive\_forest\_stands}$	9.614	3.001	3.198	-	-	0.001
$PP_{productive\_forest\_stands}$ × spline(Age)	-	-	-	3.981	10.15	< 0.001

Table S3. Percentage of landscape disturbance predicted to yield a stable population of female boreal caribou ($\lambda_f = 1$), given the percentage of potentially productive stands (the forest productivity index) and the maximum level of disturbance that can be tolerated by six boreal caribou populations, together with the lower limit yielding $\lambda_f \geq 1$ at least 95 % of the time. Current disturbance levels for the last year of data that are available are provided for each population range.

Population	% of potentially productive stands	% current disturbance	% disturbance yielding $\lambda_f = 1$	% disturbance yielding $\lambda_f \geq 1$, 95% of the time
Assinica	69.0	54.0	46.6	39.6
Eastern Manicouagan	67.7	27.7	45.8	38.9
Western Manicouagan	75.8	27.6	52.3	44.1
Nottaway	21.2	24.0	18.0	2.0
Pipmuacan	84.7	81.1	60.2	48.5
Témiscamie	60.7	44.0	40.5	33.1
